# Dual viscosity mixture vehicle for intratympanic steroid treatment modifies the ROS and inflammation related proteomes

**DOI:** 10.3389/fphar.2023.1081724

**Published:** 2023-01-19

**Authors:** Jin Woo Jung, Hui Li, Jung Hun Lee, Yu-Jung Hwang, Kisoon Dan, Moo Kyun Park, Dohyun Han, Myung-Whan Suh

**Affiliations:** ^1^ Proteomics Core Facility, Transdisciplinary Research and Collaboration, Biomedical Research Institute, Seoul National University Hospital, Seoul, South Korea; ^**2**^ Department of Otorhinolaryngology-Head and Neck Surgery, Seoul National University Hospital, Seoul, South Korea; ^3^ Transdisciplinary Department of Medicine and Advanced Technology, Seoul National University Hospital, Seoul, South Korea

**Keywords:** proteomics, perilymph, intratympanic steroid, ROS, inflammatory response

## Abstract

Until recently, the most standard treatment for sensorineural or sudden hearing loss, which is caused by inner ear damage or deterioration, has been systemic oral steroid administration. In recent, intratympanic steroid injections such as dexamethasone have been used for the treatment of sudden hearing loss as well. It is injected into the tympanic cavity through its membrane and is expected to diffuse over the round window located between the tympanic cavity and the inner ear. However, in clinical situations, the delivery time of steroids to the inner ear is shorter than 24 h, which does not allow for a sufficient therapeutic effect. Therefore, we applied a previously invented dual viscosity mixture vehicle (DVV) for intratympanic dexamethasone to a guinea pig model, which could reduce the side effects of systemic steroid administration with sufficient dwelling time for the treatment of hearing loss, and we investigated the physiological changes with a global proteomic approach. In this study, we extracted perilymph in three different conditions from guinea pigs treated with dexamethasone-embedded DVV, dexamethasone mixed in saline, and control groups to compare proteomic changes using tandem mass spectrometry analysis. After liquid chromatography coupled tandem mass spectrometry (LC-MS/MS) analysis, we first identified 46 differentially expressed proteins (DEPs) that were statistically significant after one-way ANOVA multiple-sample test. We also performed pairwise comparisons among each group to identify DEPs closely related to the treatment response of dexamethasone-embedded DVV. Gene ontology enrichment analysis showed that these DEPs were mostly related to inflammation, immune, actin remodeling, and antioxidant-related processes. As a result, the proteome changes in the DVV-treated groups revealed that most upregulated proteins activate the cell proliferation process, and downregulated proteins inhibit apoptosis and inflammatory reactions. Moreover, the reactive oxygen process was also regulated by DEPs after DVV treatment.

## Introduction

Intratympanic (IT) dexamethasone delivery is now a standard of care treatment for sudden hearing loss. Firm Level A and B evidence has been accumulated that supports the efficacy of IT dexamethasone in hearing recovery. According to the most recent guidelines from the American Academy of Otolaryngology (Chandrasekhar et al., 2019), IT dexamethasone is a Level A recommendation in patients who have not fully recovered after receiving first-line treatment.

However, there are several problems with the current IT dexamethasone treatment. Due to drainage through the Eutachian tube (Patel et al., 2019), the drug/vehicle that has been injected into the middle ear only lasts for several hours, at most. The short duration of the drug action may limit dexamethasone’s therapeutic outcome. Also, the mechanism of dexamethasone in the inner ear is poorly understood. The cochlea is covered with thick bone, and the inner ear compartment is isolated from other parts of the body by the blood labyrinthine barrier (LBL). Only a handful of studies have been able to access inner ear fluid for in-depth proteomic analysis (Szeto et al., 2021; Durisin et al., 2022; van Dieken et al., 2022).

Our group recently reported a beneficial effect of an IT dual viscosity mixture vehicle (DVV) in hearing recovery (Li et al., 2021). Thanks to the high viscosity and adhesive property of DVV, the drug/vehicle is less likely to drain through the Eustachian tube and lasts much longer in the middle ear, serving as a potent drug depot. While saline was drained within 1 day, the DVV lasted for at least 49 days in the middle ear (Li et al., 2021). The hearing outcome was significantly better in the DVV group (35.3–38.3 dB SPL) compared to the saline (42.5–63.4 dB SPL) and control (60.5–72.0 dB SPL) groups. Morphological evaluation of cochlear hair cells also supported this finding. There were significantly more hair cells in the DVV group (28.2–68.2 outer hair cells/200 µm) than in the saline (4.6–57.1 outer hair cells/200 µm) or control (0.2–42.0 outer hair cells/200 µm) groups. Despite encouraging outcomes, the exact mechanism of DVV in better hearing has not been reported. Dexamethasone is a well-known drug that is related to wound healing, antioxidant process, and reducing inflammation. However, no study has been able to prove whether this mechanism of action is also true in the inner ear when delivered *via* IT DVV.

Collectively, we observed the proteome changes within the efficiency of dexamethasone-embedded DVV treatment using MS-based proteomics technology, which was applied in a previous study (Lee J. H et al., 2021). Therefore, our hypothesis was that proteins that are related to ROS and inflammation would be greatly modified in the DVV group, since dexamethasone lasts longer in the middle ear compared to the saline group. The goal of this study was to observe the proteome changes within the efficiency of dexamethasone-embedded DVV treatment using MS-based proteomics technology.

## Materials and methods

### Experimental animals and preparation of the drug/vehicle

This study was approved by the Animal Research Committee of Seoul National University Hospital, and all animal care was supervised by the Institutional Animal Care and Use Committee Institute (IACUC 18–0168-S1A0). The detailed methods on experimental animals and drug/vehicle preparation are described elsewhere (Lee J. H et al., 2021; Li et al., 2021). In brief, Albino Hartley guinea pigs (Cavia porcellus) weighing 300–350 g were used (22 ears). Perilymph was collected twice, 2 μL each with a time interval of approximately 30 s. When the first sample was drained from the round window, it took approximately 10 s for the perilymph to refill the ST enabling the second sampling. The ears from guinea pigs were categorized into three groups, according to the treatment strategy. The first two groups were treated with the same concentration (12 mg/ml) of IT dexamethasone phosphate; however, the vehicles for delivering dexamethasone differed between the two groups. Dexamethasone was mixed inside the high and low molecular weight hyaluronic neuronic acid (HA) in the DVV group (n = 10), whereas in the saline group (n = 7), the vehicle was physiologic saline. The control group (n = 5) did not receive any treatment. Therefore, 22 samples were harvested from 22 ears of 15 animals (Table 1). For each animal, the left and right ears were allocated in such a way that individual animal-associated variability was minimized. The specific composition and preparation process of the drug/vehicle are reported elsewhere (Kang et al., 2020; Li et al., 2021). In brief, dexamethasone disodium phosphate (water-soluble salt form) was prepared in 10 mM phosphate buffer at a concentration of 1.2% (w/v). Low-molecular-weight HA (10–100 kDa, MNH Bio Co. Ltd. Hwaseong-si, Korea) was completely dissolved in the solution at a concentration of 1.0% (w/v) to coat the drug. Dexamethasone mixed in low molecular-weight HA was prepared. High-molecular-weight HA (5,000–10,000 kDa, MNH Bio Co. Ltd.) was dissolved in the dexamethasone-low-molecular weight HA solution by vigorous mixing until a concentration of 2.0% (w/v) was reached. Hyaluronic acid was filtered with a 0.2 μm cellulose acetate membrane to prevent bacterial and mold contamination. As for the dexamethasone sodium phosphate, the total aerobic microbial count was less than 103 cfu/g, total combined mold and yeast count was less than 102 cfu/g, and bacterial endotoxins were less than 0.25 EU/mg. Loading the drug in the vehicle was performed in a sterilized container and environment.

**TABLE 1:** Demographics of the 22 subjects included in this study.

Sample number (analysis number)	Sample name	Ear	Group	Volume (ul)	Grade
5	10–2	R*	Control	2	1
7	12–1	L*	Control	2	2
11	13–2	R	Control	2	1
37	14–2	R	Control	2	1
41	16–4	R	Control	2	1
15	12–1	R	Saline	2	1
17	12–2	R	Saline	2	1
45	14–3	R	Saline	2	1
47	15–1	R	Saline	2	1
49	15–2	R	Saline	2	1
51	16–2	R	Saline	2	1
53	16–3	R	Saline	2	1
23	11–1	L	DVV	2	1
27	12–2	L	DVV	2	1
33	13–3	L	DVV	2	2
57	14–2	L	DVV	2	1
59	14–3	L	DVV	2	1
61	15–1	L	DVV	2	2
65	15–3	L	DVV	2	1
67	15–3	R	DVV	2	1
69	16–1	L	DVV	2	1
71	16–2	L	DVV	2	1

*R = Extracted from right side of ear.

*L = Extracted from center side of ear.

### Intratympanic drug delivery

The detailed methods on IT drug delivery are described elsewhere (Lee J. H et al., 2021). Animals were anesthetized with 2 mL/kg of xylazine (Rompun, Bayer-Korea, Seoul, Korea) and 1.2 mL/kg of zoletil (Zoletil 50, Virbac, Seoul, Korea) using intramuscular administration. IT dexamethasone administration was performed under a surgical microscope (OPMI Pico, Carl-Zeiss, Oberkochen, Germany). An Angiocath Plus 24-gauge needle (BD, Sandy, UT, United States) was connected to a 1-ml syringe (KovaxSyringe 1 ml, Korea Vaccine Co., Seoul, Korea) with a miniextension tube (Mini-Volume Line, Insung Medical, Seoul, Korea). An air vent was first made in the anterior superior quadrant of the tympanic membrane (TM). The needle was inserted carefully and the drug/vehicle was injected at a low speed. The injection was stopped when the drug/vehicle completely filled the middle ear (bulla) or the drug/vehicle leaked out through the air vent. The volume of the drug/vehicle injected into the middle ear cavity was similar 40–60 μL between the DVV group and saline group. After injecting the drug/vehicle into one ear, the other ear was also immediately injected with a different drug/vehicle; the time interval between the first and second injections was <5 min. To prevent position effects, the animals were placed in a straight prone position, without leaning toward the left or right side, until the experiment was concluded. The drug/vehicle that was injected, and the order in which the drug/vehicle was injected, were randomized.

### Perilymph sampling

The detailed methods on experimental animals (Lee J. H et al., 2021) and drug/vehicle preparation are described elsewhere (Li et al., 2021). Perilymph sampling was performed under anesthesia. After retroauricular incision, skin and fibromuscular layers were removed for verifying the landmark structure under an operating microscope. Round cutting burr was used to open the bulla and expose the round window. Without damaging the round window membrane, the bone dust and tissue fluid were cleared with paper gauze from the bulla and round window niche. The collection of perilymph was performed 3 days after IT injection. This time delay was determined based on our previous publication (Li et al., 2021), considering a significant hearing difference that was found between 1 and 4 days. A 10 μL LongReach Pipette Tip (Multimax, USA) with distal end core diameter ranging from 480.65 to 640.87 μm was introduced slowly into the round window. The round window was not ruptured until the final sampling moment. After sampling the perilymph very slowly with a micropipette (Eppendorf Research plus, Eppendorf, Germany) which was set at the volume of 2 μL, the volume of perilymph was double checked with a separate gauge. Purity of perilymph was screened (from grade 1 to grade 4), by comparing the colour of the sample to a standardized colour map.

### Sample preparation for proteome analysis

The guinea pig perilymph sample preparation of DVV group (n = 10), capsulated dexamethasone within DVV (n = 10), saline mixed dexamethasone treated (n = 7), and non-treated group (n = 2) was performed as described previously with some modifications (Lee J. H et al., 2021). The perilymph sample was extracted from one ear or both ears of the guinea pig. Among them, the perilymph with sufficient amounts were extracted twice, while those with insufficient amounts were extracted only once. The protein digestion process was optimized to 2–5 μL of perilymph samples. Digestion buffer (8 M UREA, 5 mM tris [2-carboxyethyl] phosphine, and 20 mM chloroacetamide in 50 mM ammonium bicarbonate) was added to the perilymph sample for a brief period. The mixture was boiled for 30 min at 60°C to denature and alkylate proteins. Trypsin digestion was performed at 37°C overnight using a trypsin/LysC mixture at a 100:1 protein-to-protease ratio. All resulting peptides were acidified with 10% trifluoroacetic acid and desalted using homemade C18-StageTips as previously described (Lee J. H et al., 2021). Desalted samples were completely dried with a speed-vac and stored at −80°C.

### LC-MS/MS analysis

The data-dependent acquisition methods were conducted with an Ultimate 3000 UHPLC system (Dionex, Sunnyvale, CA, United States) coupled to a Q-Exactive HF-X mass spectrometer (Thermo Scientific, Hamburg, Germany) as previously cited modifications (Han et al., 2014; Lee et al., 2018). The digested peptides were re-suspended in 0.1% formic acid after C-18 desalting procedure (Han et al., 2014). Peptide samples were separated on a two-column system with a trap column and an analytical column (75 µm * 50 cm) with 120-min gradients from 2% to 32% acetonitrile at 300 nl/min. The column temperature was maintained at 60°C using a column heater. The column eluent was delivered to the Q-Exactive HF-x *via* nanoelectrospray. For label-free quantification, a survey scan (350–1,450 m/z) was acquired with a resolution of 70,000 at m/z 200. A top-20 method was used to select the precursor ion with an isolation window of 1.2 m/z. The MS/MS spectrum was acquired at a high collision dissociated-normalized collision energy of 30 V with a resolution of 17,500 at m/z 200. The maximum ion injection times for the full and MS/MS scans were 20 and 100 m, respectively.

### Data processing for label-free quantification

All MS raw files were processed by using interface of MaxQuant (version 1.5.3.1) (Tyanova et al., 2016a). MS/MS spectra were searched from the UniProt protein sequence data set (December 2014, 88,657 entries) using the Andromeda search engine (Cox et al., 2011). Primary searches were done using a 6-ppm precursor ion tolerance. The MS/MS ion tolerance of 20 ppm was used. Carbamido-methylation of cysteine was specified as the control modification, and N-acetylation of protein and oxidation of methionine were considered as variable modifications. Enzyme specificity was set to full tryptic digestion. Peptides with a minimum length of six amino acids and up to two missed cleavages were included. The acceptable false discovery rate (FDR) was set to 1% at the peptide, protein, and modification levels. To maximize quantification events across samples, we enabled the ‘Match between Runs’ option of the MaxQuant platform. For label-free quantification, the Intensity Based Absolute quantification (iBAQ) algorithm (Schwanhäusser et al., 2013) was used as part of the MaxQuant platform. Briefly, the iBAQ values calculated by MaxQuant are the raw intensities divided by the number of theoretical peptides. Thus, the iBAQ values are proportional to the molar quantities of the proteins.

### Statistical analysis

Perseus software (version 1.6.43.0) was used for all statistical analyses of MS data (Tyanova et al., 2016b). In case of label-free quantification, iBAQ intensities were log2-transformed. After filtering out proteins with at least 70% valid values in each group, missing values were imputed assuming a normal distribution of 0.5 width and 1.8 downshifts. The data were then normalized *via* width adjustment normalization. The multiple comparison test were performed with one-way ANOVA and *t*-test was also applied for individual group comparison. The differentially expressed proteins (DEPs) from both analysis filtered with *p*-value lower than 0.05. In the case of the individual *t*-test, the fold-change (FC) values were calculated to refine the DEPs with a union of $\log_{\;2}\left(FC\right)$ lower than −0.584963 and greater than 0.584963.

### Bioinformatics analysis

Most biological process, molecular function, and pathway analyses were performed using DAVID (Huang et al., 2009) and STRING version 11 (Szklarczyk et al., 2019). The identified biological processes were clustered using the EnrichmentMap (Merico et al., 2010) and AutoAnnotate (Kucera et al., 2016) application in Cytoscape tool (version 3.8.2) (Shannon et al., 2003). Proteins detected by both quantification methods and common interacted proteins using two different topological methods were analyzed using Venny 2.1.0 (bioinfogp.cnb.csic.es/tools/venny). Protein–protein interactions (PPIs) and visualization of protein expression with *p*-values were mapped using Cytoscape (version 3.8.2). For interactome analysis, PPI analysis were combined with STRING version 11.5 (Szklarczyk et al., 2019). The up-stream signaling or canonical pathway analysis was considered within Ingenuity and KEGG pathway analysis (Kanehisa and Goto, 2000).

## Results

### Relative quantification of Guinea pig perilymph treated with dexamethasone embedded within dual viscosity mixture vehicle

**FIGURE 1:**
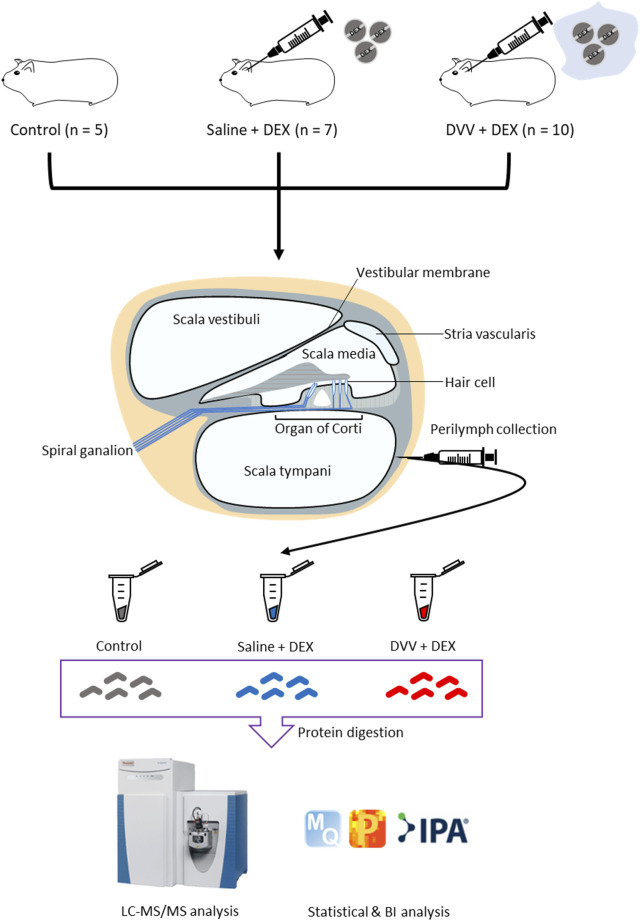
Overall scheme of proteomic analysis of guinea pig perilymph after dexamethasone treated carried by dual viscosity vesicle (DVV).

**FIGURE 2:**
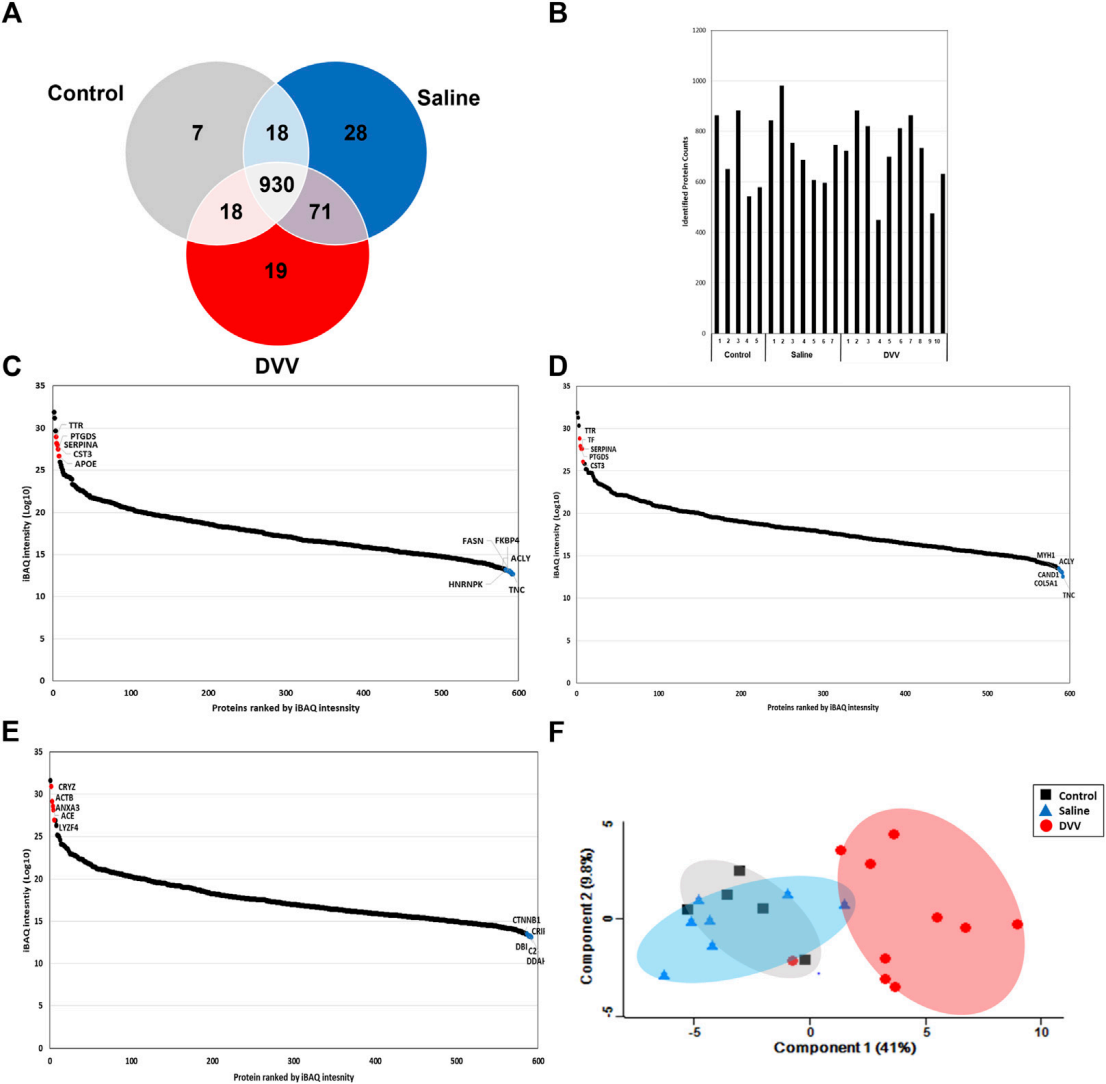
**(A)** Identified number of proteins and common DEPs from control, saline, and DVV group from LC-MS/MS analysis. **(B)** Number of identified proteins of each analyzed sample. **(C)** Dynamic range of protein abundance with top 5 up and downregulated proteins from control, **(D)** saline, and **(E)** DVV group. **(F)** The PCA plot of protein expressions from each group after width adjustment normalization.

**FIGURE 3:**
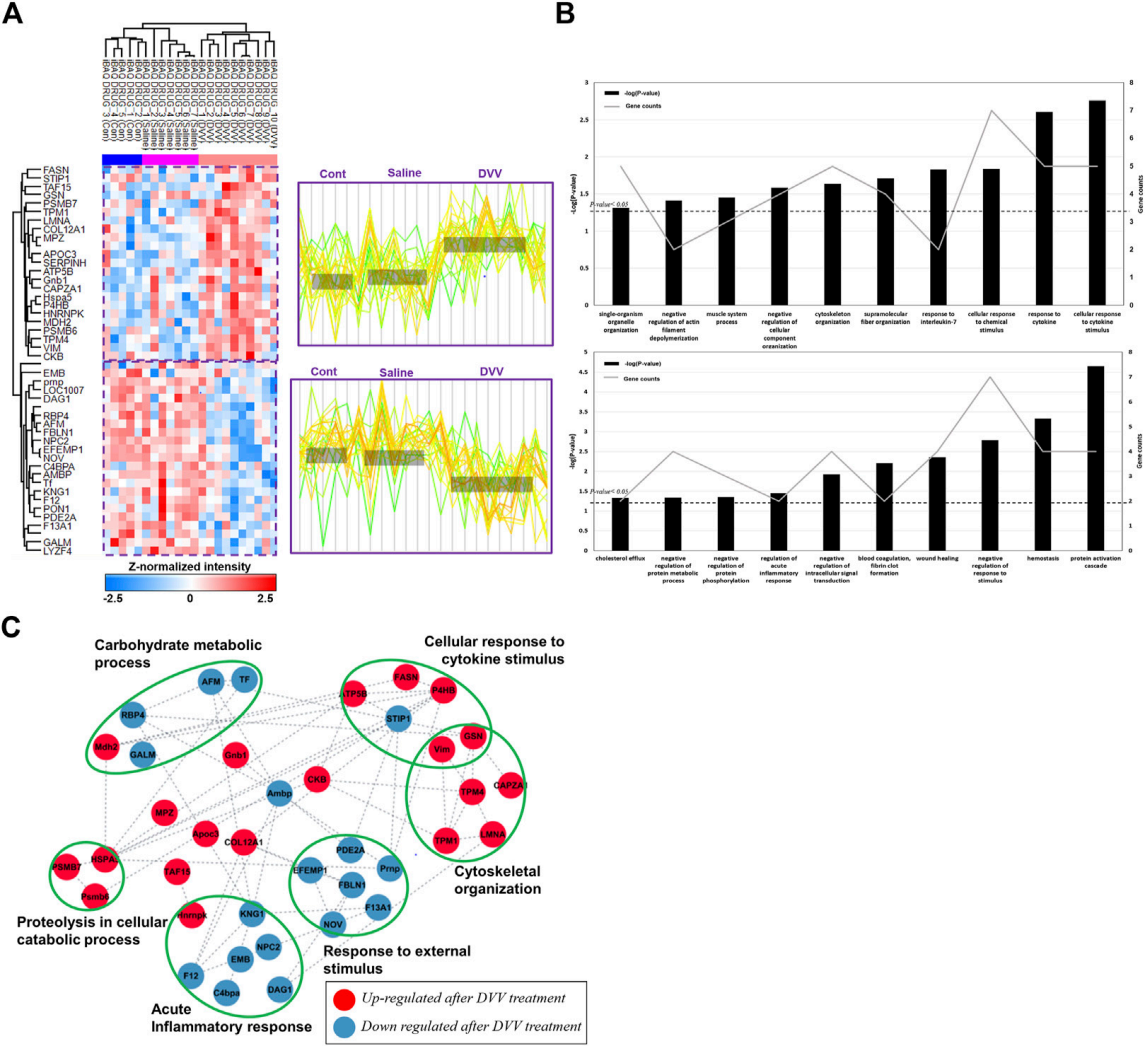
Hierarchical clustering with protein expression of DEPs and gene ontology results after LC-MS/MS analysis. **(A)** Hierarchical clustering of 46 DEPs after one-way ANOVA-test (*p*-value< 0.05).The identified DEPs consisted two specific clusters which are specifically upregulated in DVV group and downregulated in DVV group. **(B)** The biological process of DEPs that are included in two different clusters by gene ontology analysis and the number of proteins categorized in each process. **(C)** The protein-protein interaction (PPI) of DEPs, clustered with specific biological process. The red node represents upregulated proteins after DVV treatment and blue node represents downregulated proteins after DVV treatment.

Our study was designed to discover the molecular changes and clinical application of DVV carriers that embedded dexamethasone through the biological process of and related signaling pathway analysis of DEPs. Consequently, we performed global proteome profiling of guinea pig perilymphs that were treated with DVV capsulated with dexamethasone, dexamethasone mixed in saline, and a non-treated group (Figure 1). The LC-MS/MS analysis identified 973, 1,047, and 1,038 proteins from the control, dexamethasone mixed with saline, and dexamethasone carried in the DVV groups, respectively (Figure 2A). Among those, 593 quantifiable proteins that were detected more than 70% among all of the samples in each group were filtered for further processing (Figure 2B). The number of quantifiable proteins and the dynamic range of identified proteins from each group are also provided in Figures 2C–E. The protein and peptide FDRs of the identified proteins were below 1% (Q-value <0.01). Before statistical comparison, a principle component analysis (PCA) plot was presented after width adjustment normalization to explore the discrimination power by identified protein abundance. The result shows that the DVV-treated group was distinctively clustered, but the other groups were not specifically clustered, although there was a slight overlap between them (Figure 2F). To compare the protein expressions of three different groups, we performed one-way ANOVA multiple-sample comparison test and determined 46 DEPs that were statistically significant (*p*-value <0.05). Hierarchical clustering represented three specific clusters over each group. Based on protein expression patterns, each cluster was distinctly separated within two clusters that were DVV, specifically upregulated pattern and downregulated patterns, with no specific difference between the saline and control groups (Figure 3A).

### Physiological process and molecular function of specific DEPs after dual viscosity mixture vehicle treatments

The protein expression was distinctively clustered within two different patterns, as described in the colorimetric scheme presented with the expression patterns. The first cluster included the relatively upregulated proteins within DVV-treated groups compared with the saline mixed dexamethasone and control groups. The other clusters were specifically downregulated proteins within the DVV group. To determine the biological processes and physiological traits after DVV treatment, we proceeded to a gene ontology analysis of DEPs categorized in those two different clusters. The results showed that the upregulated proteins after DVV treatment were related to the response to cytokine stimulus, cytoskeleton organization, and the negative regulation of actin filament depolymerization. On the other hand, the downregulated proteins after DVV treatment were categorized in the protein activation cascade, wound healing, and regulation of the acute inflammatory response (Figure 3B). Moreover, PPI analysis was performed to examine the interaction between the overall identified DEPs. The STRING database (version 11.5) (Szklarczyk et al., 2019) was applied to plot interaction with a medium confidence of interaction score (<0.4), and functional analysis results (or visualization) were refined within the Cytoscape tool (Figure 3C) (Shannon et al., 2003). Within the interaction, some upregulated proteins after DVV treatment were clustered, and functional analysis resulted in cytoskeletal organization. Among these, many proteins acted to inhibit actin filament formation and release actin biogenesis. For instance, lamin A/C’s (LMNA) deficient cells increased myosin bipolar filament accumulation (Wiggan et al., 2020) and gelsolin (GSN) lowered actin filament formation (Feldt et al., 2018). On the other hand, the downregulated proteins after DVV treatments were classified into terms of acute inflammatory response and response to external stimulus. Interestingly, the downregulated proteins are associated with a reduction in the frequency of the immediate defensive reaction to infection or injury caused by DVV treatment. Specifically, the kininogen 1 (KNG1) knockout model decreases cytokine production and inflammatory monocytes that depress the kallikrein-kininogen system (Stadnicki et al., 1998; Wang et al., 2018). C4b binding protein alpha (C4BPA) chain is principally employed in critical inflammatory and coagulation process (Iqbal et al., 2022). Therefore, our results suggest that DVV treatment induces a decrease in the acute inflammatory response within the adaptive immune process and inhibits actin cytoskeletal organization.

### Detailed comparison between each group by protein-protein interaction

**FIGURE 4:**
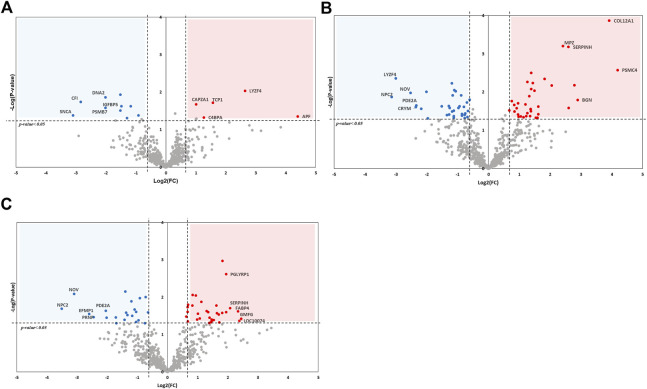
Volcano plot of DEPs after comparison between each group by t-test analysis. The x-axis of plot presented logarithmic value (log2) of fold-change between two comparing groups and dashed line indicates fold change greater than 1.5 and lower than −1.5. The *p*-value after *t*-test analysis were presented on y-axis and *p*-value lower than 0.05 is marked as dashed line. **(A)** DEPs compared with control and saline treated group. **(B)** DEPs compared between saline and DVV treated group. **(C)** DEPs compared between control and DVV treated group.

Although, one-way ANOVA test presented significant expression patterns within the DVV group, there were much similar expression patterns between control and dexamethasone mixed in saline group. Therefore, we also performed a paired *t*-test between the three different groups for a detailed comparison. The DEPs with upregulated (*p*-value <0.05 and fold-change ≥1.5) and downregulated (*p*-value <0.05 and fold-change ≤ −1.5) expressions were selected after each comparison. The MS analysis identified 16 (up = 6, down = 10), 54 (up = 32, down = 22), and 70 (up = 36, down = 34) DEPs from a comparison between the control and saline groups, the saline and DVV-treated groups, and the control and DVV-treated groups, respectively. All identified DEPs from each comparison group were respectively visualized using volcano plots (Figures 4A–C), and all of the DEPs are listed in Supplementary Tables S1–3. These statistical results revealed that there was not much difference between the control and saline groups, while protein expression comparisons between the control or saline groups and the DVV-treated group showed more remarkable changes. These results proved that dexamethasone carried by DVV treatment exhibits better therapeutic effects in pre-clinical level and suggests the possibility that DVV treatment could widely affect pathogenically than saline-mixed dexamethasone treatment, which is the traditional way in present.

**FIGURE 5:**
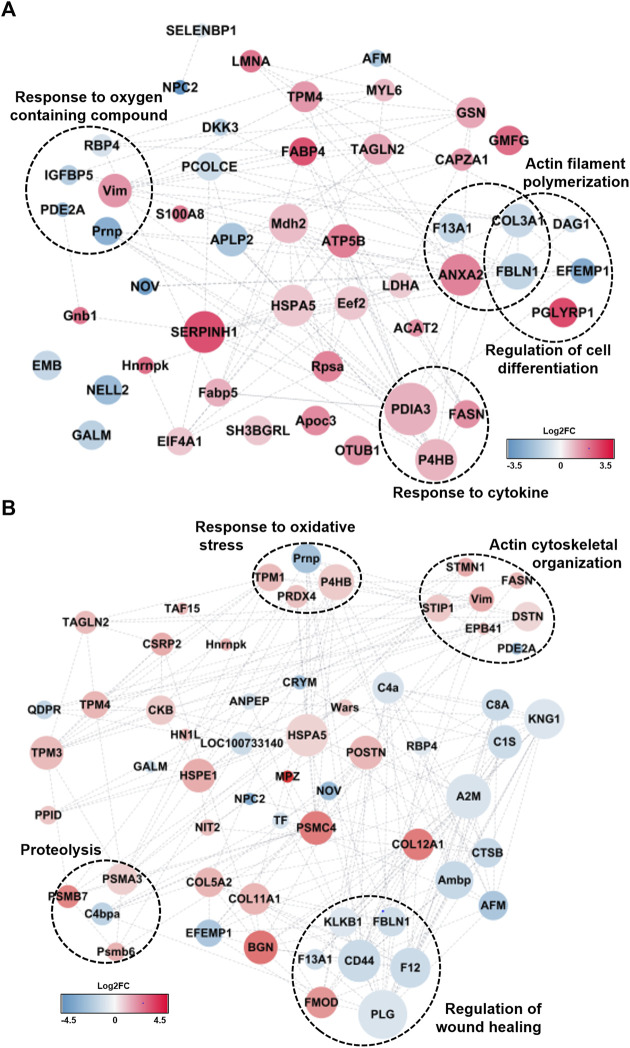
PPI analysis of DEPs after DVV treatment compared from **(A)** control and **(B)** saline group with molecular functions. The Log2FC of DEPs were represented in colorimetric scheme. The red node presented upregulated proteins after DVV treatment and blue node presented those of downregulated. The size of node exhibited the interaction score between proteins. The biological process that are equivalent from global ANOVA-test were clustered.

From the pairwise comparison between each group by *t*-test, we discovered that protein expression levels were relatively not much different between saline-treated and control groups in comparison with the difference between DVV-treated and control groups. Therefore, there are specific physiological or biological traits that could reflect such differences. To discover biological functions among different groups after DVV treatment, we performed network analysis using PPIs of DEPs from a comparison between the DVV-treated group with saline-treated and control groups. These were also embedded with protein expressions and presented as log2FC values and interaction scores. The gene ontology (GO) analysis of those were also performed to explore and compare the biological process over each group (Supplementary Tables S5, S6). The PPI plot of the comparison between the DVV group and the saline-treated group is described in Figure 5A. Consistent with the results obtained from the one-way ANOVA analysis, biological processes, such as actin filament organization or response to cytokine, are enriched in the sub-network. Furthermore, responses to oxygen-containing compounds were specifically observed in the PPI network. Interestingly, most of the proteins included in this term were downregulated after DVV treatment. The absence of vimentin (VIM), the only upregulated protein in response to the oxygen-containing compound process, induced reactive oxygen species (ROS) production and intensified bacterial killing (Mor-Vaknin et al., 2013). With the reported functions and other downregulated proteins, it is possible to propose that DVV treatment decreases the ROS production-related process or activation.

Distinct physiological processes were identified from the DEPs compared to the DVV-treated and saline-treated groups. In the PPI network, the actin cytoskeletal organization or response to oxidative stress was also clustered similarly to the comparison between the DVV-treated and control groups. Notably, proteins related to the regulation of wound healing were clustered (Figure 5B). This biological process is known to include the rate or extent of events that recover integrity to a damaged tissue. Despite almost all proteins being downregulated, fibromodulin (FMOD) was the only upregulated protein in the regulation of the wound healing process reported to repair cutaneous wounds from adult FMOD knock-out mice with the development of scar formation, block wound closure, and reduced angiogenesis. A previous study suggested that this could be caused by exogenous FMOD administration (Zheng et al., 2017). Fibulin-1 (FBLN1) affects several disease pathogenesises and related tissue remodeling. The genetic inhibition of FBLN1 decreases the inflammatory cells by reducing pro-inflammatory cytokines within chronic obstructive pulmonary disease (Liu et al., 2016) and its expression increases infiltrating macrophages by elevating the stromal and immune scores in high FBLN1 tissues (Gong et al., 2020).

### Pathway analysis after dual viscosity mixture vehicle treatments

**FIGURE 6:**
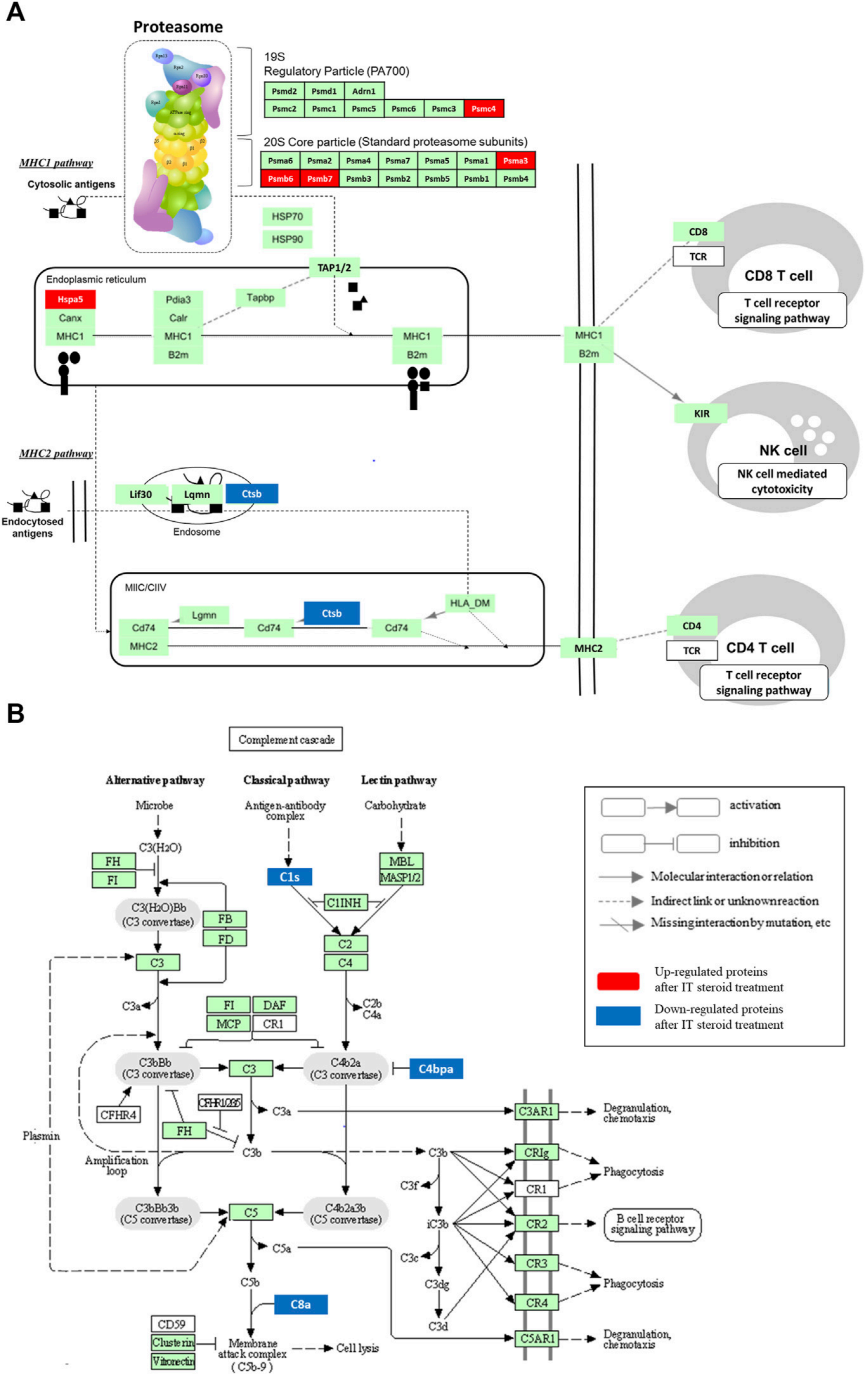
KEGG pathway description of DEPs embedded in antigen processing with proteasome complex pathway **(A)**, and complement and coagulation cascade **(B)**. Among the KEGG pathway filtered with FDR value lower than 0.05, the immune related antigen processing (map04612) coupled with proteasome (map03050) pathway, and complement and coagulation cascade (map04610) were resulted with identified DEPs.

Moreover, to investigate the molecular interaction, reaction, or related physiological pathways of identified DEPs after IT dexamethasone treatment with a DVV carrier, a KEGG pathway analysis was applied (Kanehisa and Goto, 2000). Specifically, the antigen processing and presentation pathway (map04612) and proteasome complex (map03050), which is also a part of the antigen processing pathway, were identified (Figure 6A). The cathepsin B (CTSB) protein—the downregulated protein in our study, which is categorized in the antigen processing pathway—is well known for controlling mediators of Th1 immune reaction in antigen-presenting cells (Gonzalez-Leal et al., 2014). During antigen processing, the proteasome complex transforms several subunits and forms an immunoproteasome. These immunosubunit formations adapt to serve the immune system as well (Kloetzel, 2001). The 26 S proteasome regulatory subunit 6B (PSMC4) located in regulatory particles, proteasome subunit alpha type 3 (PSMA3), and proteasome subunit beta types 6 (PSMB6) and 7 (PSMB7) included in core particles were upregulated after IT dexamethasone treatments. The proteasomes play an important role in the formation of antigenic peptides for presentation on the major histocompatibility (MHC) class Ⅰ and the activation of the immune process as well (McCarthy and Weinberg, 2015). It has also been reported that proteasome mutation, which results in loss of activation, causes autoinflammation managed by the interferon gene signature (Goetzke et al., 2021). Therefore, the identified DEPs announced increased proteasome activity after IT dexamethasone treatment and supported antigen presentation. On the other hand, the downregulated proteins in the analysis (complement C1s [C1S], C4bp alpha-chain [C4BPA], and complement C8 alpha chain [C8A]) are categorized in complement and coagulation cascade (map04610; Figure 6B). The identified DEPs are especially consisted within the classical complement pathway, which usually acts as an antigen-antibody complex formation. Specifically, the downregulation of C4BPA inhibits C3b construction by suppressing C3 convertase, and downregulation of C8a would also lower complement activation and interfere with the development of a membrane attack complex (MAC) (Oikonomopoulou et al., 2012).

## Discussion

In this study, we investigated the therapeutic effects of IT dexamethasone treatments within DVV carriers compared with the classical treatment method mixed with saline and non-treated samples. After MS analysis, the DEPs among the three different groups were identified by a multiple-sample test with ANOVA, and each group was independently examined by *t*-test. The DEPs acquired from the ANOVA test were plotted with hierarchical clustering by K-means clustering. The analyzed samples were divided into three different clusters with a *p*-value lower than 0.05. Based on the expression of DEPs, two different clusters were produced that were specifically upregulated after the DVV-treated group was compared with the saline or control group, and *vice versa*. These were further implemented for DAVID and STRING functional analysis to explore the physiological traits after IT dexamethasone treatments within DVV. The specifically upregulated proteins after DVV treatments presented a cellular response to cytokine stimulus, cytoskeleton organization, and negative regulation of actin filament depolymerization. Those of the specifically downregulated proteins were categorized as wound healing, regulation of acute inflammatory response, blood coagulation, and fibrin clot formation. After refining the redundant biological processes, we clustered the DEPs categorized with related functional terms and plotted the protein interactions using the Cytoscape tool. The individual group comparison with the *t*-test also implemented equivalent results.

The DEPs compared from each group were plotted as a volcano plot. This demonstrated that there were not many protein expression changes between the saline mixed with dexamethasone treated group and the control as compared with the dexamethasone treated within DVV and control groups. To reveal the differences affected by DVV, we also performed GO analysis and plotted it with PPI. The results showed that the DEPs were related to the response to oxygen-containing compounds or to oxidative stress and the apoptosis process. The related DEPs compared with the control group were mostly downregulated after DVV treatment. Among these, retinol binding protein 4 (RBP4) has been studied for its impact on cardiovascular disease. It has been reported that elevation of serum RBP4 induces vascular oxidative damage and increases mitochondrial reactive oxygen species (Wang et al., 2015). The inhibition of phosphodiesterase 2A (PDE2A), which is another downregulated protein, mitochondria-dependent cell death through regulation of cAMP/PKA signaling (Monterisi et al., 2017), and prevents ROS-induced vessel sprouting in mouse aortic explants *ex vivo* (Diebold et al., 2009). Although ROS-related DEPs between the DVV and saline treatment groups were upregulated, the equivalent physiological effects were covered. Among the six peroxiredoxins (PRDXs), which are potentially drug targets of antioxidant proteins, PRDX4 was upregulated after DVV treatment and localized to the mitochondria, nucleus, and endoplasmic reticulum (ER). It protects cells against oxidative stress in the ER and reduces inflammation, especially in the kidneys (Lee H. et al., 2021). Prolyl 4-hydroxylase subunit beta (P4HB), ER stress, and oxidative stress-related proteins are also upregulated proteins. Its role in this activity is to prevent programmed cell death or release ROS-induced oxidative stress (Zhou et al., 2019).

These two proteins have also been reported as the interest targets of intestinal mucosal injury (Zhu et al., 2013). Within those aspects, the actin filament or cytoskeletal organization-related DEPs were clustered and also presented inflammatory-related processes in both results from comparison between the saline and control with DVV treated group. The stathmin (STMN1) functions to regulate signals of the cellular environments and microtubule filament system, and it suppresses actin and cytoskeletal regulatory proteins by modulating phosphorylation *via* ROCK signaling (Fife et al., 2014). Moreover, it has been reported that knockdown of destrin (DSTN) in human stromal stem cells stabilizes actin filaments, and DSTN is applied as actin deplolymerizing factors (Chen et al., 2018). Likewise, most of the upregulated proteins in actin cytoskeletal-related proteins inhibit their organization. As actin cytoskeletal is a major regulator of the structural remodeling of the extracellular matrix and is important for efficient wound healing (Chen et al., 2018), we expected that these features could also be related to downregulated DEPs that are clustered in the wound healing process. One of these is the plasminogen (PLG) protein, which decreases the accumulation of fibrin during the healing of radiation wounds and enhances the anti-inflammatory effect (Fallah et al., 2020). The coagulation factor XIII A chain (F13A1) is a main substance in blood coagulation and enhances wound healing in various types of tissues by expending multiple cellular functions (Gemmati et al., 2016). Collectively, our biological process study proposed that IT steroid treatment embedded with DVV advanced the therapeutic effect of steroid treatment, and this activity is reflected by the reduction of ROS activity, inflammatory reaction, and actin filament mediated wound healing process.

In addition, identified DEPs were implemented in the KEGG pathway analysis to investigate the molecular interaction and relation networks. The results showed that DEPs were classified within complement and coagulation cascades, prion disease, proteasome, and microRNAs in cancer filtered with an FDR lower than 0.05 (Supplementary Table S7). Although the immune-related antigen processing and presentation pathway was not discovered from the KEGG analysis, it was also plotted because some DEPs were included and related to the proteasome pathway. Antigen presentation was mediated by MHC class Ⅰ and class Ⅱ molecules, which delivered peptides to the cell surface to be recognized by T cells. The 78 kDa glucose-regulated protein (HSPA5), located in MHC class Ⅰ and upregulated DEPs, regulated the expression of inflammatory, immune response genes, and ER stress (Fan et al., 2021). For efficient antigen presentation, the proteasome replaces its subunits with an immunoproteasome. The immunoproteasome was formed by 20 S core particle activation (Kloetzel, 2001), and proteasome subunits alpha type (PSMA3) and beta type (PSMB6 and PSMB7) were upregulated in our analysis. The peptides generated by those proteasomal protein substrates were transported to the ER by the antigen presentation complex (TAP). In the MHC class Ⅱ pathway, the cathepsin B (CTSB) protein—which plays the role of a pro-inflammatory molecule through activating NLRP3 inflammasome (Ma et al., 2022), was downregulated after IT steroid treatments with DVV. In addition, the DEPs related to the complement cascade pathway were also downregulated after IT steroid treatment. These complement proteins activate the immune response (Daugan et al., 2021) and inflammation. A published study reported that knockdown of C4b binding protein alpha (C4BPA) suppresses the protein expression of various interleukin proteins (Iqbal et al., 2022) a complements C8a, and mediates inflammation (Beinrohr et al., 2008). Collectively, the identified DEPs generally act to reduce inflammatory reactions and immune responses by deactivating the complement system and proteasome expression.

Therefore, it is expected that there would be some beneficial effects in the clinical advance by treating IT steroid treatments with DVV to guinea pig, which lessens the inflammatory response, ROS activity, and apoptotic process, compared with IT steroid treated within saline or the control group. In conclusion, we investigated the therapeutic and physiological traits of IT steroid treatments within guinea pig perilymph using global proteome analysis to compare steroid treatment mixed with saline, embedded in DVV, and the control group. Multiple sample comparisons were performed, and the individual groups were compared directly with each other. The DEPs were selected at a fold ratio of 1.5 and a *p*-value <0.05. To investigate the biological processes or molecular effects after steroid treatment with DVV, we performed GO and pathway analyses. The analyses revealed that upregulated proteins from multiple comparison tests between the three different groups were associated with the response to ROS, apoptosis-related signal, and wound healing correlated with actin cytoskeletal development. Moreover, we showed that these upregulated proteins inhibited the immune-related process and those modulated the inflammation related proteomes. The DEPs from the comparison between each individual group also showed equivalent biological processes. The KEGG pathway analysis also proposed that DEPs were categorized into immune- or inflammatory-related pathways. On the other hand, the comparison between the saline and DVV groups presented a much clearer difference than the comparison between the control and saline groups. Collectively, these results indicate that advanced interpretation of protein expression changes in the guinea pig perilymph after IT steroid treatment within DVV would improve the therapeutic effect and clinical understanding of IT steroid injection.

Funding: This work was supported by the Materials/Parts Technology Development Program (Development of industry process of a new hyaluronic acid, 200 Pa·s or more, of more than 20 times compared to the existing for sustained-release DDS, 1,415178889, 20,016,715) funded By the Ministry of Trade, Industry & Energy (MOTIE, Korea)

## Data Availability

The datasets presented in this study can be found in online repositories. The names of the repository/repositories and accession number(s) can be found in the article/Supplementary Material.
